# The Predictive Value of *PITX2* DNA Methylation for High-Risk Breast Cancer Therapy: Current Guidelines, Medical Needs, and Challenges

**DOI:** 10.1155/2017/4934608

**Published:** 2017-09-12

**Authors:** Michaela Aubele, Manfred Schmitt, Rudolf Napieralski, Stefan Paepke, Johannes Ettl, Magdalena Absmaier, Viktor Magdolen, John Martens, John A. Foekens, Olaf G. Wilhelm, Marion Kiechle

**Affiliations:** ^1^Therawis Diagnostics GmbH, Grillparzerstrasse 14, 81675 Munich, Germany; ^2^Department of Obstetrics and Gynecology, Clinical Research Unit, Klinikum rechts der Isar, Technische Universität München, Ismaningerstr. 22, 81675 Munich, Germany; ^3^Department of Dermatology, Klinikum rechts der Isar, Technische Universität München, Biedersteiner Str. 29, 80802 Munich, Germany; ^4^Department of Medical Oncology, Erasmus MC Cancer Institute, Erasmus University Medical Center, Wytemaweg 80, 3015 CN Rotterdam, Netherlands

## Abstract

High-risk breast cancer comprises distinct tumor entities such as triple-negative breast cancer (TNBC) which is characterized by lack of estrogen (ER) and progesterone (PR) and the *HER2* receptor and breast malignancies which have spread to more than three lymph nodes. For such patients, current (inter)national guidelines recommend anthracycline-based chemotherapy as the standard of care, but not all patients do equally benefit from such a chemotherapy. To further improve therapy decision-making, predictive biomarkers are of high, so far unmet, medical need. In this respect, predictive biomarkers would permit patient selection for a particular kind of chemotherapy and, by this, guide physicians to optimize the treatment plan for each patient individually. Besides DNA mutations, DNA methylation as a patient selection marker has received increasing clinical attention. For instance, significant evidence has accumulated that methylation of the *PITX2* (paired-like homeodomain transcription factor 2) gene might serve as a novel predictive and prognostic biomarker, for a variety of cancer diseases. This review highlights the current understanding of treatment modalities of high-risk breast cancer patients with a focus on recommended treatment options, with special attention on the future clinical application of *PITX2* as a predictive biomarker to personalize breast cancer management.

## 1. Introduction

Breast cancer is the most common malignancy in women with >464,000 new cases diagnosed in 2012 in Europe; 131,000 patients have died as a result of this disease [1]. In the Western, one in eight women will experience breast cancer in her lifetime; every fourth is younger than 55 years of age at the time of diagnosis [1]. Therapy decision in breast cancer is mainly based on the established histopathological parameters tumor size, lymph node status, histological type, and histological grade. These factors do provide information about the future clinical course of the disease (exemplified as disease-free survival, DFS, and overall survival, OS) of patients not subjected to any systemic cancer therapy. Different from that, predictive factors are biological markers, which foretell the probability of a cancer patient to respond to a specific anticancer therapy.

At present, these biomarkers include the steroid hormone receptors ER and PR, the oncoprotein *HER2*, the urokinase-type plasminogen activator (*uPA*), and the *uPA* inhibitor *PAI-1* [2] (ASCO: http://www.asco.org/practice-guidelines/quality-guidelines/guidelines, AGO: http://www.ago-online.de/en/guidelines-mamma/march-2016). According to the opinion of the St. Gallen breast cancer classification and treatment panel, *Ki-67*, a cell proliferation marker, should also be considered for therapy decision-making although no standard protocols for this analysis were defined and established so far [3, 4].

By use of molecular classifiers and gene expression signatures, invasive breast carcinomas can be further clinically categorized into at least four molecular subgroups (Table 1), which are associated with different clinical outcomes and are the basis for choosing which kind of systemic therapy should be applied, both for the adjuvant and the neoadjuvant setting [4–8]. Patients in the luminal A/B groups are generally treated with endocrine therapy but, due to biological heterogeneity in this group, quite often combined with chemotherapy (Table 1). It is estimated that 20–40% of those patients are treated cost- and side-effect-rich with chemotherapy, although endocrine therapy would be sufficient. Women with early-stage breast cancer presenting with *HER2*+ tumors (*HER2*-type) are generally treated in the adjuvant setting with the humanized antibody Herceptin® plus additional chemotherapy. Since such targeted therapy is not available for the TNBC patients, they are subject to systemic adjuvant or neoadjuvant chemotherapy. Yet avoiding not only overtreatment but also undertreatment of TNBC patients has become a major treatment issue. Therefore, TNBC-specific predictive biomarkers are urgently needed to allow for the identification of TNBC patients who will benefit from a particular systemic therapy. If not so, such TNBC patients should be offered alternative treatment plans to achieve optimal cancer treatment, thereby avoiding potentially toxic side effects.

## 2. Definition of High-Risk Breast Cancer Patients

**Figure 1:**
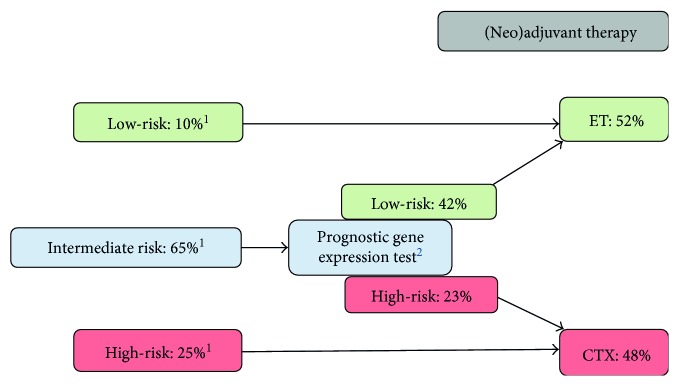
Clinicopathological risk stratification of breast cancer patients according to St. Gallen criteria. ^1^Clinicopathological risk assessment according to St. Gallen consensus panel [9, 20–24]. ^2^Endopredict®, OncotypeDX®. ET = endocrine therapy; CTX = chemotherapy.

**Figure 2:**
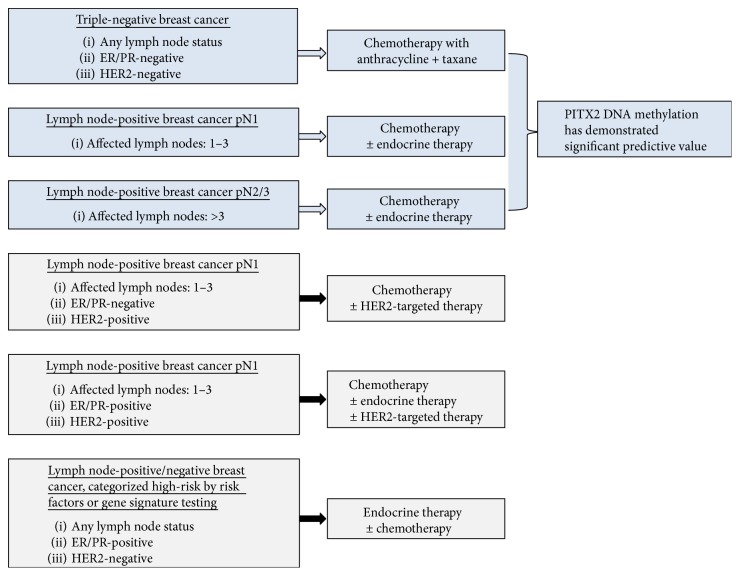
Classification of the high-risk breast cancer subtypes. *PITX2* DNA methylation status can serve as a significant predictive biomarker for anthracycline-based chemotherapy in the two major high-risk breast cancer subtypes [44, 66].

Detailed information for risk stratification of breast cancer patients who would develop metastases is urgently needed to provide effective care early enough to ensure provision of adequate anticancer treatment. In February 1998, the 6th International Conference on “Adjuvant Therapy of Primary Breast Cancer” was held in St. Gallen, Switzerland. In an attempt to solve this problem, at this conference and subsequent St. Gallen meetings, guidelines and recommendations were introduced in the scientific literature on how to select the right breast cancer patient for adequate adjuvant systemic treatment [9, 10]. According to the St. Gallen's patient stratification criteria (Figures 1 and 2), patients with a low risk to progress or to develop metastases (tumor size < 2 cm, node-negative, or up to 3 lymph nodes affected) will receive endocrine therapy if ER and/or PR are expressed. High-risk patients (node-positive with >3 lymph nodes involved, tumor size ≥ 2 cm) are subject to chemotherapy; the same applies to TNBC patients. Intermediate-risk patients, which are defined by pN0-1 [4] or histological grade 2 [11], can be allocated to the high-risk or low-risk group by applying commercially available risk-assessment tests such as MammaPrint® (Agendia), Endopredict® (Myriad Genetics), or Oncotype DX® (Genomic Health) [4, 12].

TNBC tumors and tumors with >3 affected lymph nodes are both considered high-risk breast cancers. TNBC is characterized by an increase in tumor size and high histological grade with disease recurrence as early as 3–5 years after the start of systemic cancer therapy [13, 14] and shorter survival time following the first metastatic event [1, 15–17]. Thus, the 5-year survival rate of TNBC patients is significantly reduced compared to other breast cancer subtypes.

Currently, there is no routinely available targeted therapy established for TNBC patients and no predictive factors have been introduced into the clinic which could provide information about the response of a TNBC patient to a specific cancer therapy [2]. Primary breast cancer patients with ≤3 affected lymph nodes but ER/PR negative status or *HER2* positive are considered high-risk breast cancer patients as well [18]. For those patients, anthracycline-based adjuvant chemotherapy has become the standard of care. Several studies showed that the addition of taxanes to this protocol may further improve clinical outcome. Still, not all of these high-risk patients will benefit from such an intensified therapy plan [19].

## 3. Treatment Options for High-Risk Breast Cancer Patients

In addition to the established traditional histopathological parameters (lymph node status, tumor size, histological type, and histological grade), the decision who to treat and how to treat is also based on whether ER/PR and *HER2* are expressed by the primary breast cancer tumor cells or not (Table 2). The biomarkers *uPA* and *PAI-1* are indicative factors to predict response to adjuvant chemotherapy but in node-negative breast cancer patients only [18, 25].

Originally, treatment in the neoadjuvant setting was advised for the treatment of larger sometimes inoperable breast cancers, but various considerations support the implementation of neoadjuvant systemic chemotherapy in operable breast cancer patients as well [4, 26]. Benefits of such a systemic therapy procedure are higher rates of breast-conserving surgery and the opportunity to assess early *in vivo* response to systemic treatment prior to primary surgery. In addition, this approach might improve survival of breast cancer patients by early elimination of occult metastases. Neoadjuvant chemotherapy is particularly recommended for TNBC patients and patients afflicted with *HER2*+ breast cancer [4]. Current adjuvant chemotherapy standards for early breast cancer patients involve an anthracycline plus taxane. These regimens generally do not differ for the neoadjuvant and the adjuvant settings [4].

Standard treatment for breast cancer patients with more than three affected lymph nodes is systemic therapy employing an anthracycline plus a taxane (Table 3), administered in the adjuvant or neoadjuvant setting. In addition, anti-*HER2* therapy for *HER2*+ tumors and/or endocrine therapy for ER/PR+ tumors can be applied. Results of several trials addressing N+ high-risk breast cancer patients have shown that dose-dense chemotherapy improves outcome compared to standard interval chemotherapy [4]. Patients are treated by adjuvant anthracycline-based chemotherapy; additional treatment with adjuvant endocrine therapy will be administered when tumor cells are ER/PR+. In patients with more than three affected lymph nodes, dose-dense and dose-intensified epirubicin, paclitaxel, and cyclophosphamide led not only to a significant reduction in disease recurrence and mortality but also to higher toxicities [4].

In general, in Germany, for the presurgical neoadjuvant setting, TNBC patients are treated following the AGO guidelines (http://www.ago-online.de/en/guidelines-mamma/march-2016), which are characterized by core biopsies taken to allow histological diagnosis, followed by neoadjuvant chemotherapy, primary surgery, and, if applicable, further postsurgical chemotherapy. Anthracycline-based chemotherapy is hereby the standard therapy option. For high-risk N+ TNBC patients, a definite benefit for the patients was reported when anthracyclines were applied in combination with taxanes [27]. In patients with highly proliferating TNBC, in the adjuvant setting, anthracycline-containing regimes improve disease-free and overall survival when compared to treatment with CMF (combination of cyclophosphamide, methotrexate, and 5-fluorouracile [28]).

In TNBC patients, a favorable prognosis is predicted by an effective response to (neo)adjuvant chemotherapy, which is defined by high rate of pCR (pathological complete remission) which may serve as a surrogate endpoint for estimation of long-term clinical outcomes of the patients [29]. Those patients who will not achieve a pCR are at high risk of disease recurrence within the next two years following surgery [29]. Until now, predictors of benefit from anthracyclines, or a test which predicts response to anthracycline-based treatment, are not commercially available [28]. The addition of carboplatin or cisplatin can significantly improve the rate of pCR [13]. Platinum salts do effect DNA cross-link strand breaks and, thus, prevent DNA replication [7]. Staudacher et al. [30] reported that overall survival was improved in metastatic breast cancer patients responding to platinum-based chemotherapy. Since 2014, results from several clinical trials have indicated that addition of platinum salts to a neoadjuvant anthracycline-taxane combination or sequence does improve pCR [4].

## 4. Predictive Biomarkers in High-Risk Breast Cancer

Breast cancer is a genetically and phenotypically very heterogeneous disease equipped with molecular diversity. It may exhibit with distinct clinical appearance, regarding varying response to treatment and clinical outcome. Consequently, precise breast cancer stratification for and forecasting of effectiveness of therapeutic modalities is a crucial step toward a more beneficial treatment design. For many years, breast cancer patients have been stratified according to their histopathological parameters such as histological type and grade, tumor size, lymph node status, and the *ER/PR*/*HER2*− status.

In recent years, significant advances have been made in characterizing the molecular characteristics of TNBC [5, 8, 14]. This has led to the identification of biomarkers that potentially could be used for diagnostic purpose to assess patient's prognosis or as therapeutic targets. Irrespective of the fact that today for TNBC there are no effective targeted therapies available. About one-third of the patients will achieve pCR through standard-of-care anthracycline/taxane chemotherapy; but how are these responders classified? So far, the broad molecular heterogeneity of TNBC tumors has hindered the discovery of effective biomarkers to identify such patients in order to tailor chemotherapy at an individual level [13, 31, 32]. To further characterize TNBC on a molecular level, various subtypes of TNBC have been proposed [33] (Table 4).

Eighty to ninety percent of TNBCs belong to the BL subtype. Despite some differences in the number of subtypes or the classifying methods, regarding this issue, all of the published studies suggested that TNBC consists of several subtypes and thus does require subtype-specific therapy based on its biological characteristics [34]. The clinical relevance of the TNBC subtypes listed in Table 4, however, is not yet defined. Cancer biomarkers may be useful as diagnostic, prognostic, or predictive indicators and may represent potential targets for cancer therapy [34, 35]. Although there are no clinically meaningful prognostic or predictive cancer biomarkers available, a series of potential biomarkers have been identified, for example, in the blood (*VEGF*), on the cell surface (epithelial growth factor receptor 1 (*EGFR*)), and in the cell nucleus (*BRCA1*, *BRCA2*) [32]. *VEGF* (vascular epithelial growth factor receptor) causes proliferation of endothelium cells and regulates vascular permeability and migration of endothelial stem cells from the bone marrow [34]. In TNBC patients, elevated *VEGF* levels were observed to be associated with disease recurrence and survival probability [34], making *VEGF* a potential therapy target in TNBC.

Activation of *EGFR* causes transcription of genes thus inducing cell proliferation, angiogenesis, metastatic spread, and inhibition of apoptosis. *EGFR* was found to be overexpressed in many TNBC tumors and was shown to be a prognostic factor for disease recurrence by univariate and multivariate analysis [16, 29, 32]. Currently, tyrosine kinase inhibitors (e.g., lapatinib and gefitinib) are applied in breast cancer patients to block EGF-stimulated growth signal transduction and that of *HER2* and the combination of these drugs with carboplatin or docetaxel synergistically may enhance the treatment effect in TNBC patients [29, 34, 36–38].

*BRCA1* and *BRCA2* germline mutations are more frequent in TNBC than in other types of breast cancer and affect ~30% of TNBC patients [13, 39]. There is increasing evidence that *BRCA1* germline mutant breast cancers present with above-average platinum sensitivity and increased sensitivity to poly-ADP ribose polymerase (PARP) inhibitors [13]. Germline mutations in the *BRCA* genes (*BRCA1*, *BRCA2*) predispose individuals to develop several kinds of cancer, including that of the breast [32]. Both genes play a crucial role in DNA repair processes, and the lack of functional *BRCA1/2* proteins leads to loss of repair of DNA double-strand breaks and subsequently increases the risk of cancer. Mutated *BRCA1*-related breast cancers share pathological features with TNBC, including *ER*, *PR*, *HER2*, *EGFR*, *TP53* expression, and genomic instability [34, 40].

The link between TNBC and germline *BRCA1* mutations has led to the investigation of PARP inhibitors in TNBC. PARPs are cell signaling enzymes which catalyze the poly(ADP-ribosylation) of DNA binding proteins. The main function is to act as a nick sensor for DNA damage, which plays a vital role in DNA repair through the base excision repair pathway: the base excision pathway fails when *PARP1* is inhibited. In *BRCA1*-deficient cells, inhibition of *PARP1* leads to cell death through apoptosis [29]. Trials of PARP inhibitors (e.g. iniparib, ocaparib, veliparib, talazoparib, and rucaparib) in TNBCs have shown improved rates of response and progression-free survival [6, 15, 29, 41]; however, these studies are inconclusive [7].

*Ki-67* is a marker for cell proliferation activity. In TNBC patients, *Ki-67* levels were related to tumor size and histological grade, and it was associated with increased pCR when responding to chemotherapy but with poor disease-free and overall survival [34]. *Ki-67* is one of the biomarkers which is recommended to be included in breast cancer therapy decision-making. However, so far, no standards for this analysis are defined [3].

In TNBC, the prevalence of the androgen receptor (*AR*) is ~10–20% [13, 42]. The LAR subtype of TNBC (Table 4) is characterized by luminal gene expression, enriched for AR expression and its target genes [33]. Clinically, individuals with AR-TNBC have a higher likelihood of achieving a pCR by treatment with neoadjuvant chemotherapy than those with AR+ TNBC [42]. Next-generation sequencing activities have identified further genes recurrently mutated in TNBC, including *TP53*, *PIK3CA*, *PTEN*, *RB1*, *RAS*, and *ERBB3*, but none of these genes have any predictive or prognostic relevance [31].

According to the St. Gallen risk stratification scheme (Figure 1), the intermediate-risk group (pN0-1) can be further classified into low- or high-risk by using multigene expression assays, for example, Endopredict (Myriad Genetics), Mammaprint (Agendia), OncotypeDX (Genomic Health), and others. OncotypeDX delivers a 21-gene recurrence score, which gives information about the likelihood of chemotherapy benefit as well as the risk of disease recurrence in early-stage breast cancer. This test is recommended for breast cancer patients with tumors classified as N0/N+, ER+ [12]. Mammaprint (70-gene signature) gives information about a breast cancer patient's risk for disease recurrence and identifies those patients that may safely forgo chemotherapy. Mammaprint is recommended for pN0 and pN+ breast cancer patients. Endopredict provides information how to devise personalized treatment plans for breast cancer patients. It detects the likelihood of late metastases (>5 years) and can thus guide treatment decision for CTX and predicts disease recurrence. This test is recommended for pN0-1, *ER*+, *HER2*− breast cancer patients [4, 36].

All these multigene assays, however, deliver prognostic information only but have no predictive value, also not for TNBC patients [2]. In summary, none of these tests can currently be recommended for predicting the response to a specific form of chemotherapy or to be prognostic for any kind of high-risk breast cancer patients [36]. In addition to tumor size and nodal status, the *ER/PR/HER2* status is currently the most important prognostic and predictive marker in high-risk breast cancer. There is still a so far unmet need for individualized systemic treatment of breast cancer to predict the necessity, efficacy, and potentially toxic side effects of anticancer drugs and clinical outcome of breast cancer patients under therapy. Thus, molecular tests, which can predict response or failure to a certain therapy, are highly needed to provide a tailored therapy for the appropriate patient.

## 5. Epigenetics and Breast Cancer

The term epigenetics describes dynamic alterations in a cell that switch genes on and off without changes in the DNA sequence. Epigenetic modifications are reversible. Examples of mechanisms that produce such changes are DNA methylation and histone modification, each of which alters gene expression. Many cellular processes are influenced by epigenetic changes, including gene expression, cellular differentiation, genomic imprinting, and embryogenesis [43]. DNA methylation is a chemical process that adds a methyl group to DNA on the 5th position of the pyrimidine ring of cytosine. It is highly specific and frequently happens in a region in which a cytosine nucleotide is located next to a guanine nucleotide that is linked by a phosphate: a so-called CpG site. A region of several hundred CpG sites is called CpG island [41, 43–45]. Many human gene promoters are associated with CpG islands and are usually unmethylated; a few become methylated during cell development or differentiation [41, 43]. DNA methylation can inhibit the binding of transcription factors to the promoter, and consequently, methylation in the promoter region is associated with silencing of the adjacent gene due to the abrogation of transcription [43, 46].

DNA methylation plays a crucial role in the development of a variety of cancers, including breast cancer [46]. DNA hypomethylation can lead to oncogene activation and chromosome instability in tumor development [41]. The loss of methylation in CpG-depleted regions, where CpG-dinucleotides are expected to be methylated, may be associated with aberrant or inappropriate expression of some genes that could contribute to neoplastic transformation, tumorigenesis, or cancer progression [47]. Conversely, hypermethylation has been shown to inhibit tumor suppressor genes, thereby releasing cells from their normal physiological control [41, 43].

Multiple reports have suggested that determination of the methylation status of specific promoter regions can provide important information for early detection of cancer, determine prognosis, and predict the response of a cancer patient to anticancer drugs [46]. In cancer, many tumor suppressor genes and various other cancer-related genes have been found to be hypermethylated. Their biological function includes cell-cycle regulation, apoptosis, DNA repair, cellular homeostasis, cell adhesion, and cell invasion [43, 45]. Examples for hypermethylated breast cancer-associated genes are *BRCA1*, *CCND2* (cyclin D2), *ER*, *PR*, *CDH1* (E-cadherin), and many others [43–49]. The ubiquity of such epigenetic changes in cancer events through DNA methylation has led to a variety of innovative diagnostic and therapeutic strategies.

The most recent technical advances have demonstrated the great potential of DNA methylation markers as valuable tools for decision-making in the treatment of cancer patients [43, 50]. In breast cancer, DNA methylation has shown promise as a potential biomarker for early detection, therapy monitoring, assessment of prognosis, and prediction of therapy response [46]. DNA methylation markers predicting response to endocrine therapy with tamoxifen in early and metastatic breast cancer have been described [45, 50, 51]. Furthermore, the DNA methylation status of the ER gene has been suggested as a marker for treatment response in breast cancer patients receiving antihormonal therapy [52]. Association between DNA methylation levels and clinicopathological parameters was reported, confirming complex relationships between DNA methylation and *TP53* status or the ER status [43].

There have been only a few studies focusing on the investigation of the DNA methylation of certain genes in TNBC tumor tissues. For example, a DNA methylation signature relevant for TNBC patients was identified by Stirzaker et al. exploring Cancer Genome Atlas data [53]. They showed that TNBC patients with low levels of tumor DNA methylation in their gene signature had the best prognosis, and by their gene methylation signature, TNBC could be separated from non-TNBC tumors. Hafez et al. observed that *p16* (cyclin-dependent kinase inhibitor 2A), a tumor suppressor gene, which has a central function in the regulation of cell cycle activation, was frequently hypermethylated in TNBC cases, and *p16* hypermethylation was significantly increased in TNBC compared to non-TNBC [54].

Several studies have focused on the methylation status of the *BRCA1* gene, a key player in breast cancer including TNBC. TNBC breast cancer cell lines with *BRCA1* DNA methylation were more sensitive to PARP inhibitors when *BRCA1* gene was methylated [55]. TNBC patients with pCR to adjuvant chemotherapy expressed higher *BRCA1* methylation values than nonresponders [56], and the study from Xu et al. demonstrated an increased 10-year disease-free survival of 78% in TNBC patients with *BRCA1* methylation compared to 55% in patients without *BRCA1* methylation [57].

Being aware that DNA methylation is altered in breast cancer cells compared to normal breast cells and new assays that determine these changes and thus provide information about the patient's response to anti-cancer drugs need to be introduced into clinical practice in the near future. Although some genes altered by DNA methylation have been associated with response to adjuvant therapy in breast cancer patients in small exploratory studies using laboratory-developed tests, currently, for breast cancer testing, no predictive DNA methylation test is commercially available yet. This is remarkable since, in contrast to RNA and proteins, DNA is a very stable biological material that can be extracted from the same archived clinical tissue sample that is subjected to inspection by the pathologist for routine malignancy assessment.

## 6. Pathobiology of *PITX2* in Breast Cancer

*PITX2* (paired-like homeodomain transcription factor 2, also known as pituitary homeobox 2) is a transcription factor, which is involved in the morphogenesis of anterior structures, such as eyes and teeth. *PITX2* is involved in pituitary-specific gene regulation and left-right patterning during embryonic and organogenic development [58, 59]. *PITX2* has three different isoforms, leading to three different proteins (PITX2A, PITX2B, and PITX2C), which differentially regulate transcription of their target genes [60]. Two promoters (P1 and P2) are operational: P2 drives transcription of two mRNA variants, leading to the PITX2A and B proteins; P1 drives the third transcript variant encoding the C protein [58, 61, 62]. Expression of isoforms 1 and 2 is regulated by the Wnt/*β*-catenin pathway, and expression of isoform 3 is regulated by *TGF-β* family members [48, 58]. Both the Wnt/*β*-catenin pathway and the *TGF-β* pathway play important roles in carcinogenesis, but the results from Pillai et al. support the notion that *PITX2* plays a role in mediating invasiveness of cancer cells through the Wnt/*β*-catenin pathway [58]. *PITX2* functions in the Wnt-signaling pathway by recruiting and interacting with cytosolic *β*-catenin, the central molecule in the canonical Wnt pathway. This leads to stabilization of *β*-catenin, which then enters the nucleus and associates with transcription factors leading to transcription of cell cycle regulatory and proliferation genes (e.g., *cyclin D1*, *c-Myc*, and *MMP7*), and subsequently enhances cell proliferation [17].

Even today, the role of *PITX2* in breast carcinogenesis is unclear; however, there is a role of *PITX2* DNA methylation for the prognosis of the course of the breast cancer disease. Nimmrich et al. reported that *PITX2* DNA methylation assessed in breast cancer tissue is a high-risk indicator of disease recurrence in N0 *ER/PR+* patients [48]. Another study, employing immunohistochemistry for *PITX2* determination, revealed a significant association between *PITX2* protein and *ER/PR* expression, indicating that *PITX2* and *ER/PR* protein expression may be useful prognostic markers in invasive breast cancer [63]. Jezkova et al. observed hypermethylation of *PITX2* in about 50% of invasive breast cancers [64]; an association of *PITX2* expression with established parameters such as *ER*, *PR*, and *HER2* was described by Rahman et al. [63]. Further, *PITX2* DNA methylation rates were found to be higher in tumors with elevated *ER* levels [49]. Jezkova et al. found that *PITX2* DNA methylation status was associated with high tumor grade and clinical tumor stage of breast cancer patients [64].

A further breast cancer study (*ER/PR*+, N0) was conducted by Nimmrich et al. [48]. The authors reported that *PITX2* DNA methylation acts as a statistically independent prognostic marker for these untreated breast cancer patients, implying that tumors with a hypermethylated *PITX2* status are more aggressive. In a univariate survival analysis, the authors did show that *PITX2* DNA methylation is associated with early distant metastases and poor overall survival. In a multivariate analysis, *PITX2* retained its statistical significance, together with the established prognostic factors age, tumor size, and nuclear grade, plus *ER/PR* [48]. This study also confirmed that in clinical samples *PITX2* DNA hypermethylation is positively associated with breast cancer disease progression [45, 48].

Results from the scientific literature provide further evidence that *PITX2* DNA methylation analysis may allow clinically relevant risk assessment in tamoxifen-treated primary breast cancers [51, 65]. In the study performed by Maier et al., *PITX2* DNA methylation showed the strongest correlation with metastasis-free survival in N0 *ER/PR*+, tamoxifen-treated breast cancer patients, with high *PITX2* DNA methylation representing poor metastasis-free survival [51]. Harbeck et al. could show that *PITX2* methylation in N0 *ER/PR*+, tamoxifen-treated patients added significant information to the histopathological factors tumor size, histological grade, and patient age [65]. Both studies underline that *PITX2* DNA methylation may be a potential biomarker for predicting outcome in patients with N0 *ER/PR*+ breast cancer patients treated with tamoxifen monotherapy [51, 65]. Furthermore, a strong correlation between *PITX2* DNA methylation and disease recurrence was found: 86% of patients with low *PITX2* DNA methylation were metastasis-free after 10 years, compared to only 69% with elevated *PITX2* DNA methylation [51]. In survival analyses, *PITX2* DNA methylation added statistically significant independent prognostic value to the clinical impact of the established clinical and histomorphological factors.

## 7. Clinical Implication of *PITX2* in High-Risk Breast Cancer Patients

According to the St. Gallen risk stratification panel, breast cancer patients with >3 affected axillary lymph nodes and tumor size > 2 cm are classified high-risk and treated with chemotherapy; the same applies to TNBC patients. Anthracycline-based chemotherapy has become the standard of care for these patients.

### 7.1. High-Risk Breast Cancer of the Triple-Negative Subtype (TNBC)

In a recent study, *PITX2* DNA methylation status and its clinical impact for TNBC patients were investigated [66]. *PITX2* DNA methylation was determined in primary tumor tissues obtained from TNBC patients before treatment with adjuvant anthracycline-based chemotherapy. In this retrospective study, *PITX2* DNA methylation was the only significant factor as assessed by univariate and multivariate survival analysis; in combination with *PITX2* DNA methylation status; none of the established clinical and histomorphological parameters (age, histological grade, tumor size, and lymph node status) showed statistical significance for predicting the rate of disease-free or overall survival [66].

**Figure 3:**
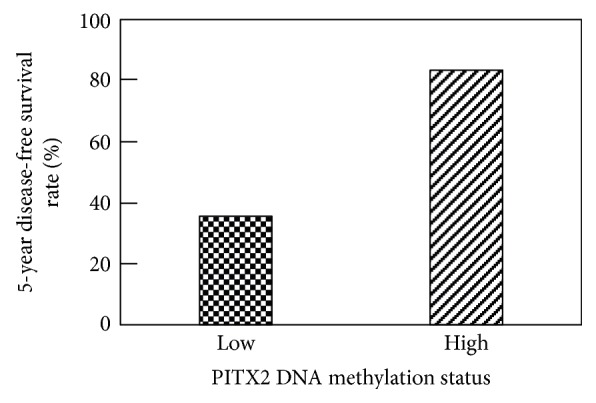
5-year disease-free survival rate analysis of TNBC patients treated with anthracycline-based adjuvant chemotherapy. At 5 years of follow up, the TNBC patients were grouped according to their *PITX2* DNA methylation value with a cut-off of 6.35 percent methylation ratio (PMR). Low *PITX2* DNA methylation status (*n* = 18) shows a poor disease-free survival rate at 5 years (35.6%); high *PITX2* DNA methylation status (*n* = 38) is associated at 5-year observation time with favorable disease-free survival (83.5%). (*p* values: log-rank test, *p* = 0.006; Wilcoxon test, *p* = 0.003) [66].

In contrast to the above-cited studies involving *PITX2* DNA methylation status in *ER/PR*+ breast cancer patients, the results obtained by Absmaier et al. revealed evidence that for TNBC patients treated with adjuvant anthracycline-based chemotherapy, a low *PITX2* DNA methylation status is associated with a decrease in the progression-free interval [66]. Kaplan-Meier analyses demonstrated that a high *PITX2* DNA methylation status is associated with a favorable prognosis for both disease-free and overall survivals. 5-year observation rates (disease-free survival) differ significantly in favor of the high *PITX2* DNA methylation group (Figure 3). For patients who did not receive any chemotherapy or who received chemotherapy that was not based on anthracyclines, this risk group separation was not apparent. These results indicate that for TNBC patients treated with adjuvant anthracycline-based chemotherapy, assessment of the *PITX2* DNA methylation status may serve as a marker that predicts response to anthracycline-based chemotherapy.

### 7.2. High-Risk Breast Cancer with >3 Axillary Lymph Nodes

**Figure 4:**
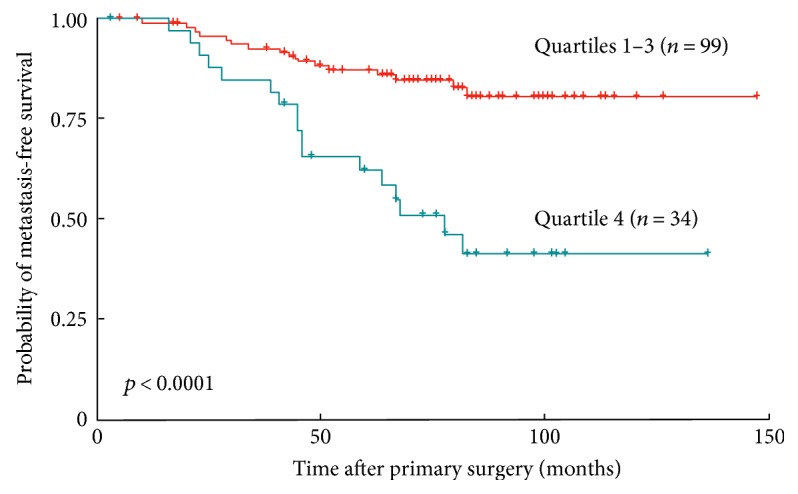
Kaplan-Meier survival curves demonstrating metastasis-free survival probability of high-risk breast cancer patients (*n* = 133). *ER*+ breast cancer patients with >3 lymph nodes affected were treated with anthracycline-based chemotherapy plus endocrine therapy. Patients were grouped according to their *PITX2* DNA methylation score. *PITX2* high gene methylation (quartile 4) predicts poor survival and *PITX2* low gene methylation (quartiles 1–3) favorable survival (data reanalyzed from [44]).

*PITX2* DNA methylation was also analyzed in high-risk (N+, *ER/PR*+, and *HER2*−) breast cancer patients treated with adjuvant anthracycline-based chemotherapy, which improved clinical outcome of the patients. In this study, the authors could show that *PITX2* DNA methylation status significantly predicts the outcome of the patients [44]. The finding was that *PITX2* DNA methylation status and that of fourteen other methylated genes predicted clinical outcome in these patients. *PITX2* DNA hypermethylation was associated with a high risk of developing metastases in this group of patients (Figure 4). In multivariate analysis, *PITX2* hypermethylation evolved as a significant marker to predict outcome [44], when assessed together with the parameters age at the time of surgery, tumor stage, nuclear grade, progesterone receptor status, and adjuvant endocrine therapy. This study provided additional strong evidence that *PITX2* DNA methylation status may serve as a useful biomarker in high-risk N+ breast cancer to predict response to anthracycline-based chemotherapy (Figure 4).

Anthracyclines can cause severe side effects; therefore, a marker which could predict sensitivity to anthracyclines in high-risk breast cancers would be highly valuable. Several studies described a positive association of *PITX2* DNA methylation status with aggressiveness of the disease and with clinical outcome. Summarizing the clinical impact of *PITX2* DNA methylation in high-risk breast cancer patients, there is evidence that *PITX2* DNA methylation may serve as a valuable predictive marker to distinguish between responding and nonresponding patients (Figure 2).

## 8. Hypothesis to Explain the Controversial *PITX2* DNA Methylation Status in Predicting Therapy Response in Breast Cancer Patients

**Figure 5:**
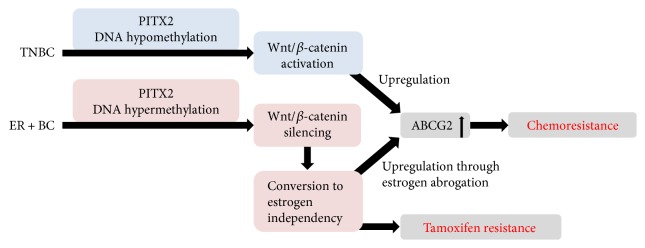
Predictive significance of *PITX2* gene methylation in high-risk breast cancer patients. TNBC and *ER*+ BC are two biologically different high-risk breast cancer entities. Yet, for both, the *PITX2* DNA methylation status has been shown to predict response or failure to anthracycline-based chemotherapy [44, 66]. The controversial results of the favorable clinical significance of *PITX2* gene hypermethylation in TNBC patients versus *PITX2* gene hypomethylation in *ER*+ BC patients are outlined. The hypothesis presented is based on published evidence; other, so far unknown, mechanisms may be involved as well. *ER*+ BC = estrogen receptor-positive breast cancer.

The data above raise the question how we can explain that hypomethylation of *PITX2* predicts nonresponders in TNBC and hypermethylation of the *PITX2* gene predicts nonresponders in *ER*+ breast cancer. The canonical Wnt-signaling pathway is activated in several tumor types including breast cancer (Figure 5). The major effector of this pathway is *β*-catenin, which is stabilized in the cytoplasm, translocates to the nucleus, and controls gene expression [17]. *PITX2* and *β*-catenin pathways upregulate the ABC transporter system, especially ABCG2, which is also known as BCRP (breast cancer resistant protein). ABCG2, which belongs to the family of membrane proteins, possesses an ATP-binding cassette and transports specific substrates actively through cellular membranes. It mediates the efflux of drugs and contributes to multidrug resistance in cancer. Substrates of ABCG2 include anticancer drugs such as anthracyclines, topoisomerase inhibitors, tyrosine kinase inhibitors, and antimetabolites [67].

The increased expression of such transporters on plasma membranes results in an increased efflux and decreased intracellular accumulation of many anticancer drugs, ultimately leading to multidrug resistance [67]. Several transcription factors regulate ABCG2, including, but not limited to, the steroid hormone receptors *ER/PR* and estrogen/progesterone response elements [67]. In TNBC, low methylation (hypomethylation) of the *PITX2* gene predicts poor disease-free and/or overall survival in nonresponders to neoadjuvant chemotherapy [58]. *PITX2* regulates the Wnt/*β*-catenin pathway [60], and *β*-catenin is required for the tumorigenic behavior of TNBC [21]. *PITX2* and the *β*-catenin pathway upregulate the ABCB1 transporter [68, 69], another efflux transporter of the same gene family involved in multidrug resistance. ABCB1 is responsible to efflux small drugs such as anthracyclines and therefore mediates chemoresistance [70]. In summary, hypomethylation of *PITX2* may lead to the activation of the Wnt/*β*-catenin pathway with subsequent upregulation of the ABCG2 or ABCB1 transporter triggering resistance to chemotherapy.

In high-risk *ER*+ BC, high *PITX2* gene methylation (hypermethylation) predicts poor disease-free and overall survival [44, 71]. The Wnt/*β*-catenin pathway is a positive regulator of *ER* in breast cancer. Hypermethylation of *PITX2* results in silencing of the Wnt/*β*-catenin pathway with subsequent downregulation of estrogen and its receptor *ER*, causing estrogen deprivation/independency in *ER*-positive cancer cells [72]. The ABCG2 transporter is downregulated by estrogen [73]; that is, estrogen deprivation leads to increased ABCG2 expression. If *PITX2* is hypermethylated, the Wnt/*β*-catenin pathway is silenced and subsequent *ER* downregulation leads to (a) overexpression of the ABCG2 transporter and thereby to anthracycline resistance and (b) tamoxifen (*ER* antagonist) resistance because of estrogen independency.

## 9. Prospects and Perspectives for Clinical Use of *PITX2* DNA Methylation Status in Cancer Patient Management

Determination of the DNA methylation status of certain genes reflects an emerging field of cancer biomarkers. Promising recent results highlight its potential for early detection, assessment of prognosis, prediction of therapy response, and therapy monitoring in various cancer diseases, which can be performed on tissue samples and on cell-free-circulating DNA in body fluids [88]. For optimum management of cancer patients, however, accurate and highly significant prognostic and predictive factors are of eminent medical need. In particular, predictive cancer biomarkers are needed to determine the right systemic therapy for patients afflicted with any of the heterogeneous high-risk breast cancer subgroups, characterized by varying outcomes, since certain systemic adjuvant therapies may be beneficial for a subgroup of patients only, while others will suffer from potentially toxic and unnecessary therapy-related side effects [41].

In order to be of clinical use, a cancer biomarker should be detectable in biological samples readily available without interrupting the routine clinical workflow. A suitable assay for routine diagnostics must be robust, simple to use, standardized, evaluated in external quality assurance schemes, and made available at affordable costs. Evaluation of such a marker should adhere to the REMARK/BRISQ criteria [79, 80]. In this respect, assessment of the *PITX2* DNA methylation status in primary tumor tissues has demonstrated its prognostic and predictive value for several cancer indications (Table 5). A cancer patient's *PITX2* DNA methylation status can be determined reliably by real-time PCR technology employing DNA extracted from formalin-fixed, paraffin-embedded (FFPE) tissue [46]. For this purpose, a validated *PITX2* test kit is required to expedite its clinical utility. Once these requirements are met, determination of *PITX2* DNA methylation status might become an important measure to aid clinicians in advising the optimal cancer therapy to their patients.

## Figures and Tables

**Table 1:** Traditional classification of breast cancer subgroups.

Classification	Expression	Distribution (%)
Luminal A	*ER/PR*+, *HER2*−	~65
Luminal B	*ER/PR*+, *HER2*+/−	~15
*HER2*-type	*ER/PR*−, *HER2*+	~5
Triple-negative	*ER/PR*−, *HER2*−	~15

According to [3, 5, 8] (http://ww5.komen.org/BreastCancer/SubtypesofBreastCancer.html).

**Table 2:** Current therapeutic regimens for breast cancers contain.

(i) For patients with luminal A/B tumors (*ER/PR*+, *HER2*−): endocrine therapy or chemotherapy followed by endocrine therapy. (ii) For patients with *HER2*+ tumors: chemotherapy plus anti-*HER2* therapy (e.g., with trastuzumab/pertuzumab) and, if appropriate, endocrine therapy. The combination with chemotherapy is currently recommended since by this therapy a survival benefit for the patients has been reported [4]. (iii) For TNBC patients: chemotherapy (anthracycline plus taxane) (AGO guidelines 2016: http://www.ago-online.de/en/guidelines-mamma/march-2016).

**Table 3:** Treatment options for high-risk breast cancer patients.

TNBC	>3 lymph nodes affected
Therapy: CTX	Therapy: CTX, anti-*HER2*, or ET
(i) Anthracycline plus taxane (ii) Neoadjuvant treatment (presurgical) (iii) Adjuvant treatment (postsurgical) (iv) Addition of carboplatin may improve pCR and event-free survival	(i) Adjuvant treatment with anthracycline plus taxane (ii) Anti-*HER2* treatment if tumor *HER2*+, and/or endocrine therapy if tumor ER/PR+ (iii) Regimens may also include platinum salts (cisplatin, carboplatin) (iv) Dose-dense or dose-intensified CTX improves outcome in N+ patients

pCR: pathological complete response; CTX: chemotherapy; anthracyclines: doxorubicin, epirubicin; Taxanes: paclitaxel, docetaxel; N+: node-positive. According to AGO guidelines (http://www.ago-online.de/en/guidelines-mamma/march-2016).

**Table 4:** Subclassification of TNBC based on gene expression analysis.

Characteristics	
BL1 (basal-like 1)	Increased expression of cell cycle and DNA repair genes
BL2 (basal-like 2)	Increased expression of growth factor signaling and myoepithelial markers
M	Increased expression of genes involved in epithelial-mesenchymal-like transition and growth factor pathways
MSL	Decreased expression of genes involved in proliferation (mesenchymal stem cell-like)
IM	Immune cell processes, expression of genes involved in cytokine signal immunomodulatory transduction pathway
LAR	Luminal gene expression and androgen receptor signaling genes

According to Lehmann et al. [33] and Szekely et al. [13].

**Table 5:** Importance and clinical relevance of *PITX2* DNA methylation status for various types of cancer.

Cancer type	Adjuvant treatment	Clinical endpoint	Clinical impact analyzed	Reference
Breast cancer (*n* = 345) LN−, *ER/PR*+	Tamoxifen	MFS	Therapy response prediction	Maier et al., 2007 [51]
Breast cancer (*n* = 412) LN−, *ER/PR*+	None	TDM, OS	Prognosis	Nimmrich et al., 2008 [48]
Breast cancer (*n* = 399) LN−, *HER2*±, *ER/PR*+	Tamoxifen	TDM	Therapy response prediction	Harbeck et al., 2008 [65]
Breast cancer (*n* = 241) LN+, *ER/PR*+	Anthracycline	DFS, MFS, OS	Therapy response prediction	Hartmann et al., 2009 [44]
Breast cancer (*n* = 100) LN±, *ER/PR*±, *HER2*±	Chemotherapy: 65% of the patients. Endocrine therapy: 39% of patients	OS, DFS, DSS	Prognosis	Buhmeida et al., 2011 [74]
Breast cancer (*n* = 149) LN±, *ER/PR*±, *HER2*±	Not disclosed	No follow-up	Association of *PITX2* with *RASSF1A* and clinicopathological parameters	Jezkova et al., 2016 [64]
Breast cancer (*n* = 56) LN±, *ER/PR/HER2*−	Anthracycline	DFS	Therapy response prediction	Absmaier et al., 2017 [66]

Prostate cancer (*n* = 719)	Observation, untreated	OS	Prognosis	Ahmad et al., 2016 [75]
Prostate cancer (*n* = 476)	Observation, untreated	Biochemical recurrence	Prognosis	Bañez et al., 2010 [76]
Prostate cancer (*n* = 356)	Not disclosed	Biochemical recurrence	Prognosis	Uhl et al., 2017 [77]
Prostate cancer (*n* = 680)	Observation, untreated	Biochemical recurrence	Prognosis	Dietrich et al., 2013 [78]
Prostate cancer (*n* = 367)	Observation, untreated	Biochemical recurrence	Prognosis	Vasiljević et al., 2014 [79]
Prostate cancer (*n* = 71)	Observation, untreated	Biochemical recurrence	Prognosis	Litovkin et al., 2014 [80]
Prostate cancer (*n* = 45)	Not disclosed	Biochemical recurrence	Prognosis	Vinarskaja et al., 2013 [81]

Biliary cancer (*n* = 80)	Not disclosed	OS	Prognosis	Uhl et al., 2016 [82]

Esophageal cancer (*n* = 305)	Chemoradiotherapy including cisplatin plus 5-fluorouracil	DFS	Prognosis Therapy response prediction	Zhang et al., 2013 [83]

Pancreatic cancer (*n* = 256)	Not disclosed	OS	Prognosis	Wang et al., 2016 [84]

Head & neck cancer (*n* = 399)	Radiotherapy ± chemotherapy	OS, DFS	Prognosis	Sailer et al., 2016 [85]
Head & neck cancer (*n* = 528)	Not disclosed	OS	Prognosis	Sailer et al., 2017 [86]

Lung cancer (NSCLC) (*n* = 474)	± radiotherapy/chemotherapy	PFS	Prognosis	Dietrich et al., 2012 [87]

DFS: disease-free survival; DSS: disease-specific survival; OS: overall survival; PFS: progression-free survival; TDM: time to distant metastasis; MFS: metastasis-free survival; LN: lymph node status; ER/PR: receptors for estrogen and/or progesterone; *HER2*: human epidermal growth factor receptor 2; TNBC: triple-negative breast cancer.
